# Spatiotemporal dynamics of low-carbon technology collaboration networks and regional public health governance implications: evidence from China’s Yangtze River Delta

**DOI:** 10.3389/fpubh.2026.1830983

**Published:** 2026-05-21

**Authors:** Feng Hu, Huijie Yang, Yilin Li, Shuang Zhao, Xiaoping Wang, Zhimin Ren, Shaobin Wei, Jiahan Hu, Shaobo Yang, Haiyan Zhou, Hao Hu, Junyu Cheng

**Affiliations:** 1 Institute of International Business & Economics Innovation and Governance, Shanghai University of International Business and Economics, Shanghai, China; 2 International Business School, Shanghai University of International Business and Economics, Shanghai, China; 3 College of Business Administration, Ningbo University of Finance and Economics, Ningbo, China; 4 Institutional Affiliation School of Management, Zhejiang Gongshang University Hangzhou College of Commerce, Hangzhou, China; 5 Institute of Digital Economy and Financial Powerhouse Building, Guangdong University of Finance, Guangzhou, China; 6 College of Engineering, University of Perpetual Help System Laguna, Biñan, Laguna, Philippines; 7 Industrial Technology Research Center, Shanghai Yice Research Institute, Shanghai, China; 8 Graduate School, Nueva Ecija University of Science and Technology, Cabanatuan, Philippines; 9 School of Economics, Shanghai University, Shanghai, China; 10 Elliott School of International Affairs, The George Washington University, Washington, DC, United States

**Keywords:** carbon emission, geographic detector, low-carbon technology, public health, social network analysis, Yangtze River Delta

## Abstract

Climate change and carbon-intensive development models pose potential long-term risks to public health by exacerbating exposure to air pollution. Promoting low-carbon technological innovation through regional collaboration has become one of the critical pathways to mitigate environmental health risks and to improve public health governance. Based on enterprise low-carbon patent data in the Yangtze River Delta (YRD) from 2014 to 2023, this study integrates spatial statistical analysis, social network analysis, and geographic detector methods to systematically investigate the spatiotemporal evolution, structural characteristics, and driving factors of low-carbon technology collaborative innovation networks. The results show that: (1) The number of low-carbon innovation enterprises in the YRD has grown substantially, with obvious spatial agglomeration. The collaborative innovation network continues to expand, while regional development disparities are widening; (2) Core cities, including Shanghai, Ningbo, Hangzhou, and Nanjing, consistently maintain central positions within the network, serving as critical hubs for low-carbon technology diffusion and regional carbon governance. Meanwhile, cities such as Hefei and Suzhou have achieved a significant increase in network centrality. (3) Economic scale, financial support, technological innovation capacity, and policy investment are the core driving factors shaping network structure and evolutionary patterns, which collectively shape urban carbon emission reduction performance and environmental health improvement. The findings provide empirical evidence to strengthen innovation-driven carbon governance and promote more equitable and resilient regional health systems under the low-carbon transition.

## 1 Introduction

Carbon-intensive development patterns not only generate adverse environmental and economic outcomes but also exert detrimental impacts on public health by intensifying air pollution exposure and triggering climate-related health risks. Existing studies confirm that 98% of low-carbon transition scenarios can generate significant health benefits through three major pathways: improved air quality, increased physical activity, and optimized dietary structure. Among these, the reduction in the incidence and premature mortality of cardiovascular and respiratory diseases resulting from better air quality constitutes the primary source of the health co-benefits associated with low-carbon policies (1, 2). Furthermore, environmental degradation significantly increases the health vulnerability of socially disadvantaged groups, thereby further widening health inequities (3). Meanwhile, affected by disparities in regional development levels, environmental governance capacity exhibits significant spatial inequality, further exacerbating the unfair distribution of health risks across regions (4, 5). Accordingly, improving carbon mitigation capacity serves not only as a core objective of climate governance policies but is also widely recognized as a vital pathway to safeguard public health security and advance health equity. Against the global backdrop of climate change and low-carbon transition, China has accelerated its “dual carbon” strategy, in which low-carbon technological innovation serves as a key engine for emission reduction and green development (6–8). However, uneven regional innovation capacity has created gaps in carbon governance and environmental governance across regions, further leading to spatial inequalities in public health risks. As a nationally strategic region, the Yangtze River Delta (YRD) faces typical challenges of unbalanced low-carbon innovation and health governance. Exploring the spatiotemporal dynamics and collaborative networks of low-carbon innovation enterprises in the YRD can not only enrich innovation research but also provide empirical support for regional green development and public health governance.

Research on enterprise spatial distribution typically employs microdata such as the number and locations of multinational enterprises, knowledge-based enterprises, and high-tech companies to analyze the evolution of their spatial patterns and influencing factors. The findings indicate that most such innovative enterprises tend to cluster in megacities, drawn to regions offering interfirm innovation collaboration, development zones, urban industrial structures, and abundant innovation resources (9–22). The fragmentation of intercity innovation activities restricts the large-scale spatial diffusion and application of technologies, and may further exacerbate the imbalance of regional environmental and health governance capacity. Existing studies mainly focus on spatial distribution and its economic effects, while paying insufficient attention to cross-regional innovation collaboration and structure evolution, especially from a public health perspective.

Innovation plays a dual role in regional environmental and health governance. On the one hand, it provides technical support for carbon emission reduction and realizes mitigation at the production end through the research and development (R&D) of low-carbon technologies. On the other hand, regional policies can optimize the spatial layout and collaborative structure of innovation networks, thereby improving the overall efficiency of cross-regional technological collaboration. Owing to such dual attributes, low-carbon technology innovation networks serve as a critical intermediate carrier connecting regional carbon governance policy interventions and regional technology development (23). Existing research on innovation networks typically employs data such as patent cooperation or transfers, corporate venture capital collaboration records, research paper collaborations, and city-level patent application volumes (24–27). By employing social network analysis and gravity models, studies have examined the spatial patterns, evolutionary mechanisms, and influencing factors of regional innovation networks. Using cities, urban clusters, or nations as research units, these investigations reveal that innovation networks exhibit small-world characteristics, pronounced spatial clustering, and core-periphery spatial patterns, with multidimensional proximity emerging as a key determinant of innovation network performance (28–44).

In terms of low-carbon technological innovation, existing studies mainly measure its development level through indicators such as R&D investment, the number of academic publications, and patent counts (45–49). Findings indicate a notable increase in the number of participating countries and scholars, with China making particularly prominent contributions (50–52). Furthermore, existing literature has reported that low-carbon technological innovation activities present pronounced spatial clustering characteristics, while innovation at the enterprise level generally suffers from insufficient endogenous motivation. Although green innovation helps enhance firms’ market competitiveness, it is still constrained by high innovation costs and uncertain future returns. In contrast, key external driving factors, including government regulation and intervention, local environmental regulations, regional environmental awareness, and average enterprise innovation capabilities, can effectively compensate for the lack of endogenous innovation incentives among enterprises (51, 53–64).

Building on the above discussion, a clear mechanism can be established linking collaborative low-carbon innovation networks to public health. First, from the perspective of innovation network theory and regional innovation systems, intercity collaboration networks facilitate the diffusion of low-carbon technologies by enhancing knowledge spillovers, improving connectivity among innovation actors, and facilitating the recombination of distributed technological knowledge across regions (39–44). Second, the enhanced diffusion and application of such technologies contribute to improved carbon-reduction capacity and more efficient environmental governance, particularly through cleaner production processes and energy structure optimization (23, 45–49). Third, improvements in environmental quality, particularly air quality, are recognized as the primary channel through which low-carbon transitions generate health co-benefits (1–3, 5). Finally, improved air quality reduces population exposure to pollutants, thereby lowering the incidence of respiratory and cardiovascular diseases and enhancing overall public health outcomes (2, 3). Therefore, the structure and inclusiveness of low-carbon innovation networks constitute a significant, yet underexplored, institutional pathway through which technological development influences regional environmental governance capacity and health equity.

In summary, existing studies rarely integrate enterprise spatial patterns and innovation collaboration networks into a unified analytical framework, and there is a lack of firm-level micro-research focused on specific technological fields such as low-carbon technologies. Statistical data show that China has achieved remarkable progress in the field of green and low-carbon technologies in terms of technological advancement, innovative entities, and overseas patent layout, gradually emerging as an important participant and driving force in the global low-carbon transition^1^. From a domestic perspective, the YRD serves as a core growth pole for high-quality economic development in China and undertakes the national strategic mission of environmental governance and integrated green and low-carbon development^2^. The regional innovation collaboration network can be regarded as a type of informal carbon governance infrastructure. By promoting knowledge spillovers, sharing emission-reduction costs, and accelerating the intercity diffusion of technologies, it plays a vital role in improving regional carbon-reduction efficiency and environmental governance capacity. Therefore, identifying the evolutionary dynamics of the low-carbon technological innovation system in the YRD not only yields theoretical innovation significance but also provides valuable implications for other regions to advance low-carbon transition and regional health governance.

The innovation of this study lies in two aspects. First, in terms of research methods and data, most existing studies adopt only a single approach, either spatial statistics or social network analysis, and primarily conduct analyses based on cross-sectional urban data. Within a unified analytical framework, this study integrates spatial statistics, social network analysis, and the geographic detector (Geodetector) method. Based on long-term low-carbon patent data from 2014 to 2023, it characterizes the spatial pattern and network structure of low-carbon technological innovation, thereby systematically revealing the dynamic evolution process and driving mechanisms of low-carbon innovation (35–43). Second, in terms of research content and analytical perspective, this paper breaks through the limitations of a single dimension of geographical space or relational networks. Taking low-carbon innovation enterprises as the research object, it integrates the dual perspectives of “geographical space and relational network”. It explores how collaborative networks shape differentiated regional carbon emission reduction performance and environmental health risks by influencing technology diffusion and resource allocation. This further expands the interdisciplinary research boundary between low-carbon innovation research and public health governance (16–22).

## 2 Research methods and data

### 2.1 Research methods

This study constructs a comprehensive analytical framework integrating spatial statistical analysis, social network analysis, and the Geodetector method. First, spatial statistical methods are employed to examine the spatiotemporal distribution and clustering characteristics of low-carbon innovation enterprises. Second, based on intercity collaboration data, social network analysis is conducted to construct innovation networks and capture their structural features, including the roles and positions of different cities. Third, building on the identified spatial patterns and network structures, the Geodetector model is applied to examine the key drivers of network formation and evolution, as well as their spatial differentiation. This integrated framework establishes a clear stepwise analytical procedure that links spatial patterns, network structures, and underlying mechanisms.

#### 2.1.1 Spatial statistical methods

ArcGIS software was used to analyze and calculate the spatial autocorrelation, spatial hotspot detection, and standard deviation ellipse analysis of low-carbon technology innovation enterprises in the YRD. This enabled an in-depth examination of the spatiotemporal pattern evolution characteristics of low-carbon technological innovation in the region (65–70).

#### 2.1.2 Social network analysis

Gephi software was employed to analyze and calculate network attributes such as network density and average degree, as well as city node attributes, including weighted degree centrality and betweenness centrality. Weighted degree centrality reflects a city’s connectivity and level of participation within the innovation network, illustrating its level of activity and influence in collaborative innovation. Betweenness centrality characterizes a city’s intermediary and bridging role within the network, reflecting its pivotal position in facilitating cross-regional technology diffusion and resource flows. This method enabled an analysis of the spatiotemporal pattern evolution characteristics of the low-carbon technology collaborative innovation networks (71–75).

#### 2.1.3 Geographical detector

In this study, the weighted degree centrality and betweenness centrality of the 2023 YRD low-carbon technology collaborative innovation network are employed as dependent variables. The reason for selecting cross-sectional data from 2023 for geographic detector analysis is primarily based on three considerations. First, 2023 marks the endpoint of the study period and can reflect the relatively stable structure of the low-carbon technology cooperation network following long-term evolution. Second, compared to earlier stages, the network connections in 2023 are more robust, and their structural characteristics are clearer, which helps enhance the reliability of identifying driving factors and avoids interference caused by structural fluctuations during the intermediate stage. Third, this time, the cross-section more accurately reflects the current context of the low-carbon transition and regional governance, thereby giving the research conclusions strong practical significance. And the choice of weighted degree centrality and betweenness centrality is motivated by their complementary roles in characterizing network structure. Two indicators characterize node features from the dimensions of “participation intensity” and “structural function,” respectively, providing a relatively comprehensive reflection of a city’s status and role within the network. Furthermore, as both indicators are continuous variables, they are suitable for the geographical detector model, thereby enhancing the robustness of driver identification. The influencing factors of the network from three dimensions: technological innovation, economic and financial, and local policy and infrastructure (4, 76–79).

### 2.2 Research data

Low-carbon technology innovation enterprises refer to those whose innovation activities center on or significantly incorporate low-carbon technologies. Given the relative difficulty in obtaining corporate financial data such as internal R&D expenditures, low-carbon technology patents serve as the most direct and measurable evidence of an enterprise’s low-carbon technological innovation output. Therefore, this study first utilizes the patent classification codes for low-carbon technologies published by the OECD’s ENV-TECH (80). A total of 103,876 patents filed in 2014, 2017, 2020, and 2023 in China, with applicants classified as enterprises, and matching the low-carbon patent classification codes were retrieved from the Incopat patent database. Second, the list of patent applicants is compiled, and the registered location, industry classification, and enterprise attribute of each applicant are matched in Qixinbao. The matching strictly follows the principle of one-to-one correspondence between the full name of the patent applicant and the full industrial and commercial registered name of the enterprise, ensuring the accuracy of patent-firm matching. For large diversified enterprises, patents are included if they fall within the predefined low-carbon technology categories, to ensure that the analysis focuses on the relevant technological activities of enterprises rather than their overall patent portfolios. A comprehensive list of 40,889 low-carbon technology innovation enterprises across 41 cities in the YRD has been identified and organized. Third, we identified 37,159 patents with more than one applicant, where only enterprise-type applicants are included. In calculating patent collaborative innovation, if a patent was filed by three applicants (A, B, and C), we counted each of the following collaborations: A–B, A–C, and B–C. This process ultimately yielded the low-carbon technology collaborative innovation network for the YRD from 2014 to 2023.

## 3 Spatiotemporal evolution of low-carbon technology innovation enterprises in the YRD

### 3.1 Temporal evolution characteristics of low-carbon technology innovation enterprises in the YRD

Analysis of the temporal changes in the number of low-carbon technology innovation enterprises in the YRD from 2014 to 2023 reveals a pronounced upward trend across all four provinces and municipalities. Jiangsu Province exhibited the most significant growth, increasing from 849 enterprises in 2014 to 10,958 in 2023, ranking first in terms of both total enterprise count and growth rate. Growth in the other three provinces/municipalities was relatively stable, reaching 4,569, 1,998, and 2,217 enterprises, respectively, by 2023. This growth trend aligns closely with China’s ongoing ecological civilization initiatives and dual-carbon goals, particularly following the 2015 Paris Agreement and the 2020 dual-carbon target announcement, which provided a strong policy impetus for the low-carbon technology sector. In terms of development stages, all provinces experienced their first explosive growth in 2017, with a significant surge in newly added enterprises. This aligns with the policy orientation of strengthening green development outlined in China’s 13th Five-Year Plan. Under the dual-carbon policy, the number of low-carbon innovation enterprises in the YRD increased markedly again in 2020. However, in 2023, while Jiangsu Province added nearly 10,000 new enterprises with strong momentum, Shanghai and Anhui provinces experienced slight declines.

**Figure 1:**
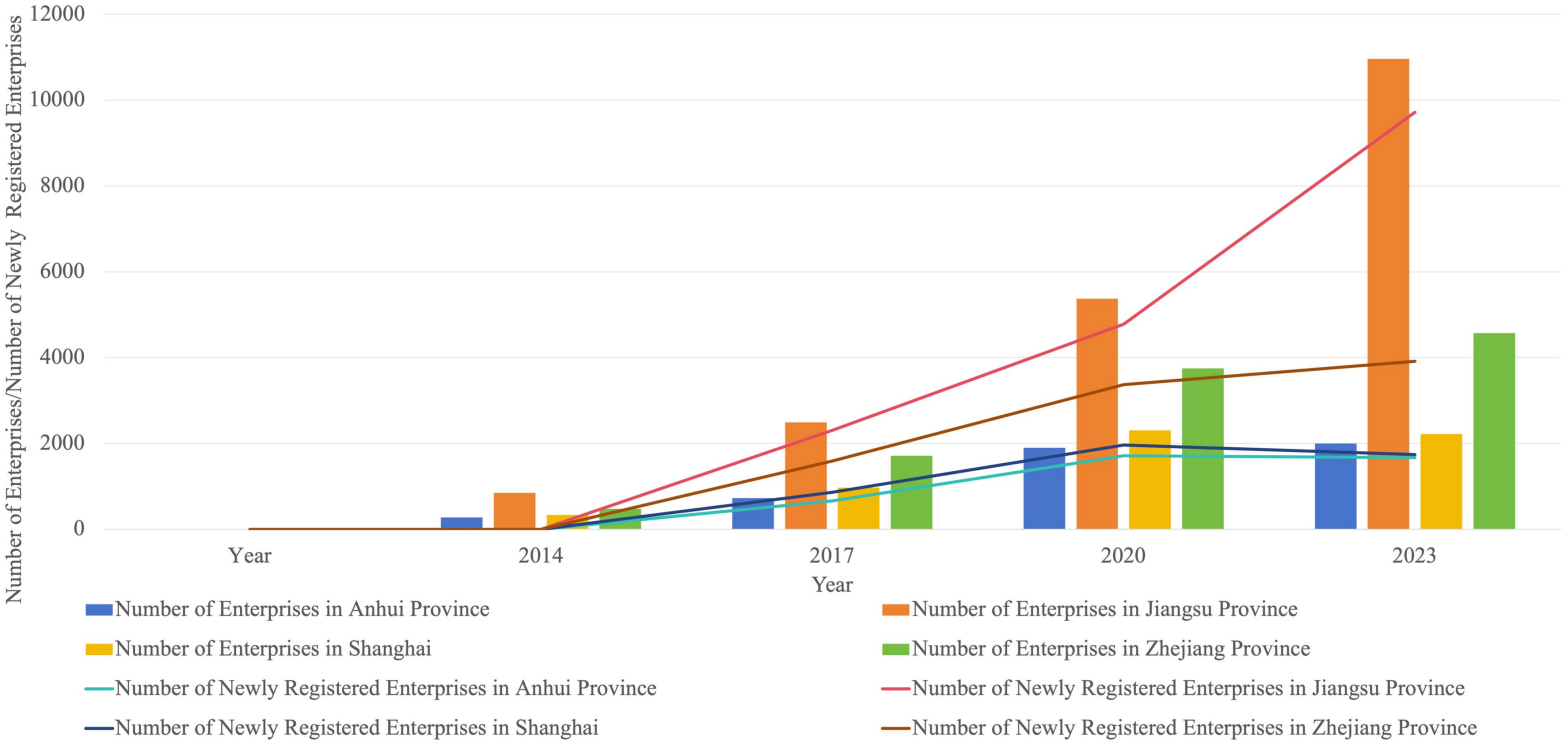
Temporal evolution of low-carbon technology innovation enterprises in the YRD, 2014–2023.

Overall, the vigorous development of low-carbon innovation enterprises in the YRD fully reflects the deep integration of national strategic guidance and regional industrial upgrading, which has not only enriched the supply of emission-reduction technologies but also accelerated the transformation away from carbon-intensive and high-pollution production models. These changes can indirectly strengthen the region’s capacity to address climate-related health risks and continuously consolidate the innovation-driven foundation for carbon emission mitigation and public health protection (see Figure 1).

### 3.2 Overall spatial distribution of low-carbon technology innovation enterprises in the YRD

The Moran’s I index for each year is significantly positive, indicating that low-carbon technology innovation enterprises in the YRD exhibit pronounced positive spatial autocorrelation in their spatial distribution, that is, a distinct spatial clustering pattern. From a temporal perspective, the clustering effect steadily strengthened between 2014 and 2017 but showed a declining trend by 2020. By 2023, the clustering effect had reached its peak, indicating that the spatial clustering trend has further intensified in recent years. Overall, these findings demonstrate that low-carbon technology innovation enterprises in the YRD have not only experienced rapid quantitative growth but also formed a spatially clustered development pattern. This provides spatial evidence for advancing the regional integration of low-carbon technological innovation networks in the YRD (see Table 1).

**Table 1:** Spatial autocorrelation analysis of low-carbon technology innovation enterprises in the YRD, 2014–2023.

Indicator	2014	2017	2020	2023
Moran’s I index	0.3250	0.3438	0.2421	0.3873
*Z* value	3.5441	3.7154	2.6897	3.8969
*p*-value	0.000	0.000	0.007	0.0000

In terms of the distribution of hot and cold spots, the spatial pattern of low-carbon technology enterprises remains generally stable, although dynamic adjustments occur in certain areas. Hot spots remain highly concentrated in the core region centered on Shanghai, including neighboring cities such as Suzhou, Wuxi, Changzhou, Jiaxing, Nantong, and Huzhou, which have advantages over others in terms of geographical proximity, industrial infrastructure, and policy coordination. Cold spots are primarily distributed in cities such as Bengbu, Bozhou, and Suzhou in northern Anhui Province, indicating a clear gradient difference in low-carbon technological innovation capacity across the YRD. Notably, some cities have undergone distinct typological shifts. Hefei has transitioned from a secondary hotspot to a cold spot, which aligns with the recent shifts in strategic focus and the realignment of innovation resources in areas such as quantum information and new energy vehicles. Zhejiang cities, including Ningbo, Shaoxing, and Taizhou (Zhejiang), have shifted from secondary hotspots to secondary cold spots, which can be attributed to the concentration of innovation resources in Hangzhou and the gradual transformation of local industrial structures. Moreover, Yancheng has moved from a subcold zone to a subhot zone, indicating improved low-carbon innovation competitiveness in northern Jiangsu. This improvement is influenced by Jiangsu Province’s coastal green development policies and targeted support for the new energy industry. Adjustments in cold and hot spots reveal the dynamic shifts in low-carbon technology R&D. Combined with subsequent regional correlation analysis, this will enable a more comprehensive assessment of a city’s low-carbon technology intensity, thereby providing support for addressing urban public health issues through technological governance.

**Figure 2:**
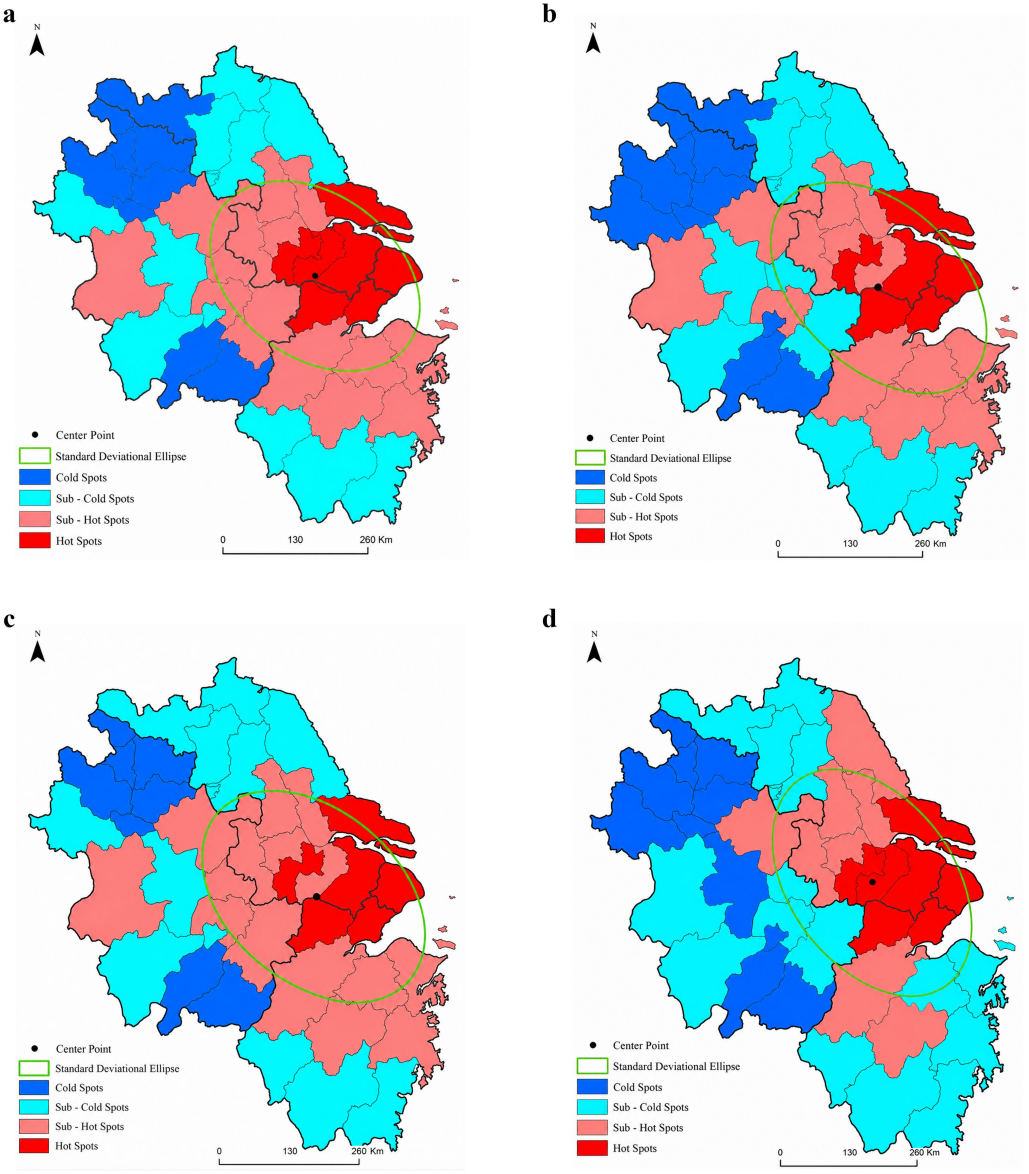
Spatial distribution of low-carbon technology innovation enterprises in the YRD, 2014–2023. **(a)** 2014; **(b)** 2017; **(c)** 2020; **(d)** 2023.

Standard deviation ellipse analysis indicates slight fluctuations in the ellipse center. Except for 2017, when it was located in Suzhou, the center remained in Wuxi for all other years, demonstrating the relative stability of the geographical focus for low-carbon technological innovation in the YRD. The standard deviation of the major axis first increased but then decreased, increasing from 204.75 kilometers in 2014 to 221.95 kilometers in 2017, but declining again to 216.65 kilometers in 2023. The angle of rotation of the ellipse steadily increased from 125.77 degrees in 2014 to 140.40 degrees in 2023, indicating that spatial clustering underwent a dispersion-to-aggregation process. The primary axis direction gradually rotated counterclockwise from northeast–southwest to southeast–northwest. The expansion and contraction of the ellipse indicate that, in the early stages, low-carbon innovations spread outward, helping to narrow regional disparities in carbon governance and health risk prevention capabilities. In the later stages, resources concentrate in the core areas, and core cities can leverage their higher network transmission efficiency to radiate outward and drive development, while this might widen disparities in technology supply. Meanwhile, this shift has accelerated the development and application of clean energy, such as wind power and marine energy in coastal areas and northwestern Anhui, thereby advancing regional energy transition and public health governance (see Figure 2).

## 4 Evolution of spatiotemporal patterns in the low-carbon technology collaborative innovation network of the YRD

### 4.1 Overall network attributes

Analysis using Gephi software reveals that the low-carbon technology collaborative innovation network in the YRD expanded continuously from 2014 to 2023. The number of nodes increased from 30 to 40, whereas the number of edges increased from 64 to 227, which indicates that the network has gradually evolved from “fragmented connection” to “overall connectivity.” This transition reflects the weakening of institutional barriers among cities as well as the continuous improvement of industrial innovation platforms and cross-regional coordination mechanisms^3^, consolidating the institutional and organizational foundation for inter-firm R&D collaboration. The average degree of the network increased from 2.133 to 5.675, while the average weighted degree rose from 8.967 in 2014 to a peak of 39.432 in 2020, then declined to 26.25 by 2023. The fluctuation was influenced by the YRD integration strategy and early-stage industrial upgrading. In the later period, affected by the COVID-19 shock and policy adjustments, the network shifted from rapid expansion toward a more stable cooperation pattern. The network diameter decreased from 5 to 4, while the average path length decreased from 2.423 to 2.092, indicating a significant improvement in network structural efficiency, which can accelerate knowledge spillover and diffusion, facilitate the regional dissemination and absorption of low-carbon technologies. Concurrently, network density increased from 0.074 to 0.146, and the average clustering coefficient rose from 0.255 to 0.529, indicating that the overall network structure became increasingly clustered and stable, with gradually prominent small-world network characteristics. High network density and strong clustering may strengthen knowledge spillovers and improve collaborative innovation efficiency, but they may also consolidate the agglomeration advantages of core nodes, resulting in obvious hierarchical differentiation and path dependence within the network (26). Meanwhile, the geographical proximity of air pollution necessitates cross-administrative collaborative governance and coordinated technological advancement across regions. So, more interconnected innovation networks can indirectly help enhance the region’s capacity for coordinated carbon governance and the long-term protection of public health (see Table 2).

**Table 2:** Collaboration network attributes of low-carbon technology innovation enterprises in the YRD.

Attributes	2014	2017	2020	2023
Nodes	30	32	37	40
Edges	64	87	189	227
Average degree	2.133	2.719	5.108	5.675
Weighted average degree	8.967	14.219	39.432	26.25
Network diameter	5	5	4	4
Network density	0.074	0.088	0.142	0.146
Average clustering coefficient	0.255	0.359	0.514	0.529
Average path length	2.423	2.269	2.105	2.092

### 4.2 Evolution of network spatial patterns

Using the natural breakpoint method, cities were categorized into four tiers on the basis of weighted degree centrality and intercity collaborative innovation intensity in 2023, with this framework applied to subsequent years. From the perspective of weighted degree centrality, Shanghai, Ningbo, Hangzhou, and Nanjing consistently occupy the core position (fourth tier) of the low-carbon technology collaborative innovation network because of their strong economic and technological innovation advantages, with Shanghai maintaining the highest centrality across all years. Cities with a higher weighted degree centrality demonstrate deeper network embeddedness, as well as stronger capabilities in coordinating collaborative innovation and absorbing external knowledge. Ningbo, Hangzhou, and Nanjing follow closely, forming the network’s secondary core cities, collectively shaping the backbone of the regional innovation network. The long-term dominant position of these core cities stems from solid economic foundations, sound industrial systems, and sustained policy support. The superposition of multiple advantages endows them with a strong capacity to gather innovation resources. Cities such as Suzhou and Hefei experienced significant increases in centrality in 2023, with Hefei entering the fourth tier that year. This progress is closely supported by top-level low-carbon policy arrangements, which have provided strong institutional backing for low-carbon technology innovation enterprises. In 2022, the Suzhou Municipal Science and Technology Bureau issued the Suzhou Science and Technology Implementation Plan for Carbon Peaking and Carbon Neutrality 2021–2030^4^, while Hefei released the Hefei’s 14th Five-Year Plan Implementation Scheme for Energy Conservation and Emission Reduction^5^ in the same year. This shift also reflects the ongoing restructuring of the regional innovation network pattern and the gradual spillover of innovation factors from traditional core cities to emerging nodal cities, which conforms to the core-periphery evolution law of innovation networks.

With respect to cooperation intensity, high-level collaborations (such as Ningbo–Shanghai and Hangzhou–Ningbo) predominantly occur among these core cities, with cooperation intensity showing marked growth over time. While low-intensity collaborations primarily occur among peripheral cities. This structure not only ensures efficient knowledge circulation and technology sharing within the core layer but also generates gradual innovation spillover effects toward peripheral regions. However, it may trigger the spatial imbalance in the allocation of innovation resources and technological capabilities, thereby undermining cities’ long-term environmental and public health governance capacity. Cooperation intensity exhibits distinct hierarchical differences: collaborative innovation is expanding from the intraprovincial level to the interprovincial level, such as through the strengthening of ties between Nanjing and Ma’anshan and Hefei and between Hangzhou and Jiaxing and Jinhua. However, intraprovincial collaborations still account for a significant proportion, exemplified by Nanjing–Suzhou and Changzhou–Wuxi within Jiangsu Province and within Zhejiang Province, such as Hangzhou–Ningbo and Jiaxing–Huzhou. This finding indicates that administrative boundaries and economic geographic proximity remain crucial factors influencing collaborative innovation. This is primarily because intraprovincial innovation cooperation benefits from consistent policies, fiscal support within the same province, and administrative barriers being broken down, thereby reducing collaboration costs. In addition, lower transaction costs, stronger social embeddedness, and more mature industrial linkages within provinces further reinforce path-dependent collaboration patterns. As a result, despite the advancement of the YRD integration strategy, administrative boundaries remain a persistent constraint on cross-regional collaborative innovation.

**Figure 3:**
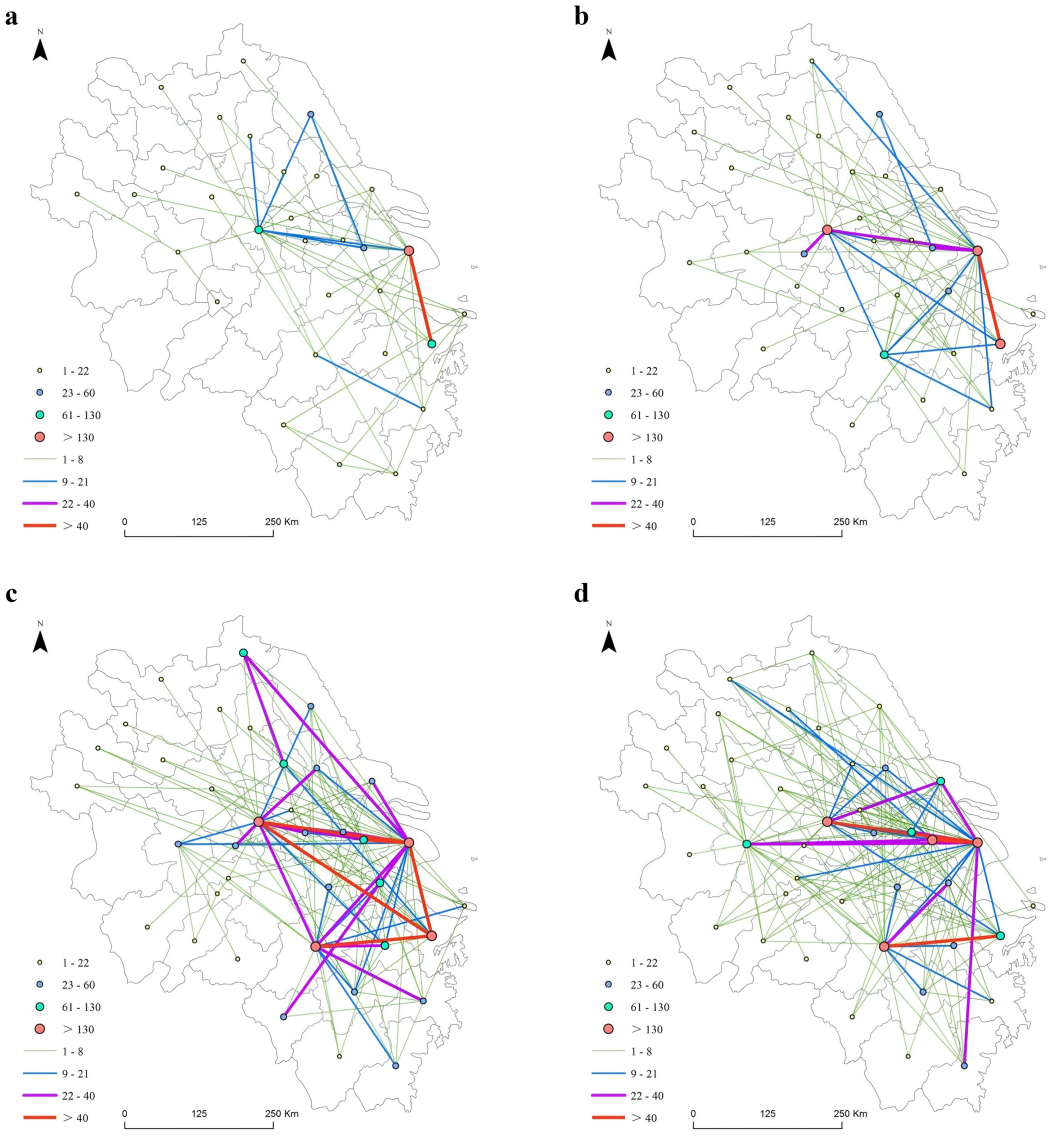
Characteristics of low-carbon technology patent transfer in China, 2014–2024. **(a)** 2014; **(b)** 2017; **(c)** 2020; **(d)** 2023.

Overall, the low-carbon technology collaborative innovation network in the YRD features a layered core–periphery structure. Shanghai, Ningbo, Hangzhou, and Nanjing form the primary core, with Ningbo, Hangzhou, and Nanjing serving as secondary cores (see Figure 3).

The betweenness centrality rankings of the YRD low-carbon technology collaborative innovation network clearly reveal that Nanjing, Shanghai, and Hefei have consistently maintained the top three positions, indicating that these provincial capital cities undertake crucial hub functions within the network and exert strong controlling power. This advantage stems from their superior top-level policy design capabilities, agglomeration of high-end scientific and technological innovation resources, mature low-carbon industrial systems, and central geographical locations in the region. In terms of evolutionary trends, Yancheng ranked fourth in 2014 but gradually dropped out of the top ten thereafter. This may stem from its initial role as a bridge between southern and northern Jiangsu because of its geographical location, a function that diminished as the network expanded. However, the transition of Yancheng to a sub-hot zone in the later stage indicates that its internal R&D activities remain active, even though its role as a bridging node within the network has weakened. Ningbo and Nantong saw significant increases in their intermediary degrees by 2023, with Nantong notably entering the top five, indicating enhanced hub functions in regional collaboration. Cities such as Taizhou (Zhejiang), Jiaxing, and Suzhou experienced ranking fluctuations but remained within the top ten. Overall, the high-intermediary cities and high-weighted degree centrality cities in the YRD’s low-carbon technology collaborative innovation network significantly overlap. Throughout its dynamic evolution, this network has been driven primarily by a few hub cities, revealing significant hierarchical disparities in cities’ capacity to reduce public health risks through low-carbon technological governance. In particular, the persistence of established diffusion pathways may further reinforce inequalities in governance capacity across regions (see Table 3).

**Table 3:** Top ten cities in the YRD by betweenness centrality in low-carbon technology collaborative innovation network.

Rank	2014	2017	2020	2023
1	Nanjing	Nanjing	Nanjing	Nanjing
2	Shanghai	Shanghai	Shanghai	Shanghai
3	Hefei	Hefei	Hefei	Hefei
4	Yancheng	Hangzhou	Hangzhou	Hangzhou
5	Hangzhou	Taizhou (Zhejiang)	Taizhou (Zhejiang)	Nantong
6	Zhoushan	Jiaxing	Suzhou	Suzhou
7	Nantong	Shaoxing	Jiaxing	Jiaxing
8	Taizhou (Zhejiang)	Ningbo	Changzhou	Ningbo
9	Suzhou	Wuxi	Huzhou	Wuxi
10	Jiaxing	Changzhou	Wuhu	Huzhou

## 5 Influencing factors of the YRD low-carbon technology collaborative innovation network

### 5.1 Variable selection

Existing scholarly research indicates that technological innovation development is influenced by dimensions such as a city’s economy, scientific and technological innovation, and infrastructure (30–35, 51, 54–61). Therefore, this study analyzes the influencing factors of the low-carbon technology collaborative innovation network in the YRD from three dimensions: scientific and technological innovation, economic and financial, and local policy and infrastructure. Within the scientific and technological innovation dimension, the following variables were selected: the number of low-carbon technological innovation enterprises, the number of patent authorizations, the technology market transaction volume, and the number of higher education institutions. For the economic and financial dimension, we selected Gross Domestic Product (GDP), year-end deposits in financial institutions, year-end loans in financial institutions, total retail sales of consumer goods, and the shares of secondary and tertiary industries in GDP. For the local policy and infrastructure dimension, we selected development zones, education expenditure, telecommunications and postal service revenue, and science and technology expenditure as influencing factors. The dependent variables are the weighted degree centrality and betweenness centrality of the 2023 low-carbon technology collaborative innovation network in the YRD. Among higher education institutions, double first-class universities were assigned a value of 2, while regular universities were assigned a value of 1. For development zones, national-level zones were assigned 2, provincial-level zones 1, with other influencing factors sourced from the China Urban Statistical Yearbook. Before geographic detector analysis, natural breakpoint methods were employed to categorize influencing factors and dependent variables into five levels.

### 5.2 Analysis results

Geodetector analysis reveals that all influencing factors are statistically significant at the 1% level or higher, indicating that a multidimensional set of factors influences the formation of the low-carbon technology collaborative innovation network in the YRD. It should be noted that the q-statistic in the Geodetector measures the explanatory power of spatial stratification, and the associated *p*-values reported in this study are used only to test the significance of the q-statistic itself.

Network weighted degree centrality reflects a city’s position within the low-carbon technology network. A higher weighted degree centrality indicates that a city has established more frequent and stable cooperative relationships with other nodes. In the economic and financial dimensions, year-end financial institution loan balances and GDP ranked first and second in weighted degree centrality q values within the YRD low-carbon technology diffusion network. This finding indicates that economic scale and financial support strongly explain a city’s position within the low-carbon technology cooperation network. A large economic scale can provide a complete ecosystem for low-carbon technological innovation, from experimentation and promotion to industrialization, thereby attracting the establishment of low-carbon technology innovation enterprises and partners. Such agglomeration effects not only strengthen local innovation capacity but also facilitate cross-regional knowledge spillovers through network connections, thereby significantly improving the city’s weighted degree centrality. Meanwhile, R&D innovation requires substantial upfront investment and entails uncertainties and risks, so long-term, stable credit further stimulates the agglomeration of innovation. The strong explanatory power of total retail sales of consumer goods further indicates that market size and economic vitality can influence low-carbon technological innovation through demand-side mechanisms. On the one hand, robust market demand provides application scenarios and commercialization prospects for clean technologies. On the other hand, stronger consumption capacity facilitates the diffusion of green technologies and faster technology transformation, thereby forming a positive circular mechanism. In terms of the technological innovation dimension, low-carbon technology innovation enterprises and patent authorization counts demonstrate strong explanatory power, indicating that cities with strong knowledge production capacity are more likely to become knowledge source nodes in the network, attracting more partners and establishing stable innovation connections. In the policy and infrastructure dimension, all relevant influencing factors exhibit explanatory power exceeding 0.7, underscoring this dimension’s crucial role in low-carbon technological innovation collaboration. Scientific and rational policy systems and infrastructure investment provide physical and institutional guarantees for innovation cooperation, thereby reducing the difficulties of regional collaborative responses to environmental governance challenges.

Network betweenness centrality reflects a city’s control level, where higher betweenness centrality is generally located in structural hole positions. By connecting originally fragmented innovation agents, cities facilitate the cross-regional flow of knowledge factors and, to a certain extent, shape the paths of technology diffusion and the direction of resource allocation. In terms of scientific and technological innovation, the explanatory power of higher education institutions and scientific research expenditure is significantly stronger than their influence on weighted degree centrality, indicating that the bridging function of the research system plays a more critical role. As core carriers of knowledge production and talent cultivation, universities are inherently endowed with cross-regional and cross-industry collaborative attributes. Meanwhile, local governments effectively reduce institutional and cost barriers to collaborative innovation by increasing scientific and technological investment, participating in industry–university–research (IUR) cooperation, and establishing joint R&D funds. This enables cities with abundant science and technology, such as Nanjing and Shanghai, to occupy a prominent hub position within the innovation network. In the economic and financial dimension, GDP and year-end financial institution loan balances retain high explanatory power. This reflects how a city’s economic and financial resources not only strengthen its position in low-carbon technological innovation cooperation but also enable it to serve as an intermediary for resource allocation and collaborative coordination. These capacities may further enhance cities’ ability to support innovation-driven carbon reduction strategies that contribute to improved environmental quality and long-term public health protection (see Table 4).

**Table 4:** Factor analysis results for factors influencing the YRD low-carbon technology collaborative innovation network.

Influencing dimension	Influencing factor	Weighted degree centrality		Betweenness centrality	
		*Q*-value	*p*-value	*Q*-value	*p*-value
Technological innovation dimension	Number of low-carbon technological innovation enterprises	0.782	0.000	0.652	0.000
	Number of patent authorizations	0.780	0.000	0.725	0.000
	Technology market transaction volume	0.676	0.019	0.674	0.021
	Higher education institutions	0.684	0.011	0.757	0.000
Economic and financial dimension	GDP	0.828	0.000	0.849	0.000
	Year-end financial institution deposit balance	0.794	0.000	0.688	0.019
	Year-end financial institution loan balance	0.864	0.000	0.820	0.000
	Total retail sales of consumer goods	0.735	0.000	0.682	0.005
	Secondary and tertiary industries as % of GDP	0.684	0.000	0.393	0.005
Local policy and infrastructure dimension	Development zones	0.701	0.004	0.624	0.041
	Education expenditures	0.722	0.000	0.697	0.005
	Telecommunications and postal service revenue	0.735	0.000	0.544	0.098
	Science and technology expenditures	0.764	0.000	0.847	0.000

Further analysis using interaction detection modules revealed that the interactions between the number of low-carbon technology innovation enterprises and other influencing factors all exhibited two-factor enhancement, indicating the crucial synergistic role of these two elements in collaborative low-carbon technological innovation. The interaction Q value between the number of low-carbon technology innovation enterprises and year-end financial institution loan balances reached 0.917, while the interaction Q value with science and technology expenditures reached 0.921. This finding indicates that the synergies of innovation entities + financial support and innovation entities + policy assistance significantly increase a city’s central position and hub function within low-carbon technology collaborative innovation networks. Low-carbon technological innovation requires substantial and sustained capital investment. As an emerging field with unstable market and profitability prospects, it must rely on policy and financial intervention and support. For instance, Shanghai has advanced its low-carbon development through top-level design by issuing the Action Plan for Accelerating Green and Low-Carbon Transformation in Shanghai (2024–2027) and the Shanghai Carbon Credit Management Measures (Trial)^6^. It has established a carbon credit platform to incentivize low-carbon development among enterprises and citizens. Furthermore, interactions between low-carbon technology innovation enterprises and higher education institutions, patent authorization counts, and technology market transaction volumes exhibit high q values (mostly exceeding 0.85). This finding indicates that IUR collaboration and technology commercialization mechanisms jointly reinforce a city’s role within innovation networks. Although the multi-dimensional driving model can significantly elevate a city’s network position, it also brings prominent unintended adverse consequences. This model inherently tends to further consolidate the first-mover advantages of core cities endowed with superior initial innovation foundations, financial resources, and policy support, exacerbating the spatial polarization of low-carbon innovation resources across the region and crowding out innovative inputs in peripheral areas. Such outcomes solidify rigid technology diffusion pathways, placing peripheral cities at a structural disadvantage in the acquisition, transformation, and practical application of low-carbon technologies. This widens inter-regional disparities in environmental governance capacity and generates spillover effects on public health, aggravating inequalities in regional health risk prevention and emergency response capabilities. Accordingly, cross-regional multi-stakeholder collaborative governance carries crucial practical significance. It can break the path dependence of intra-provincial cooperation, alleviate the above polarization effect, facilitate coordinated development between core and peripheral nodes, improve the inclusiveness and efficiency of cross-regional low-carbon technology diffusion, and ultimately contribute to the realization of regional public health equity (see Table 5).

**Table 5:** Interaction detection results between influencing factors of the YRD low-carbon technology collaborative innovation network and low-carbon technology innovation enterprises.

Influencing dimension	Interaction between the number of low-carbon technology innovation enterprises and other influencing factors	Weighted degree centrality	Betweenness centrality
Technological innovation dimension	Number of patent authorizations	0.895	0.874
	Technology market transaction volume	0.885	0.852
	Higher education institutions	0.852	0.872
Economic and financial dimension	GDP	0.869	0.874
	Year-end financial institution deposit balance	0.890	0.836
	Year-end financial institution loan balance	0.917	0.885
	Total retail sales of consumer goods	0.866	0.796
	Secondary and tertiary industries as % of GDP	0.886	0.755
Local policy and infrastructure dimension	Development zones	0.833	0.730
	Education expenditures	0.895	0.885
	Telecommunications and postal service revenue	0.872	0.766
	Science and technology expenditures	0.903	0.921

## 6 Implications for public health governance

The findings of this study provide important implications for understanding regional disparities in public health governance from the perspective of low-carbon innovation networks.

First, spatial analysis reveals a persistent core–periphery pattern in the distribution of low-carbon innovation activities. This uneven structure implies unequal access to low-carbon technologies and, consequently, uneven capacity to mitigate air pollution and climate-related health risks. Peripheral cities may face higher environmental health exposure due to limited participation in innovation networks, highlighting the need to incorporate spatial equity into public health governance. Second, the evolution of the collaborative innovation network shows increasing connectivity and efficiency in knowledge diffusion. While this facilitates broader dissemination of low-carbon technologies, the dominance of core cities suggests that diffusion pathways remain concentrated. As a result, the benefits of technological progress may not be evenly shared. Improving network inclusiveness is therefore critical for reducing regional disparities in environmental health outcomes. Third, network structure analysis highlights differentiated city roles. Cities with high weighted degree centrality concentrate innovation resources, while those with high betweenness centrality facilitate cross-regional knowledge flows. Effective public health risk mitigation thus depends not only on innovation capacity but also on diffusion efficiency. Strengthening both is essential for a balanced governance system. Finally, the Geodetector results indicate that low-carbon innovation capacity is shaped by economic strength, financial support, technological resources, and policy environments. Strong interaction effects among these factors suggest that public health governance requires coordinated, multidimensional policy interventions.

Overall, low-carbon innovation networks function as an upstream mechanism influencing environmental quality and population health. Promoting more inclusive and well-connected networks is therefore essential for achieving both effective climate governance and equitable public health outcomes.

## 7 Conclusions and policy implications

### 7.1 Conclusion

Based on low-carbon patent data of enterprises in the YRD from 2014 to 2023, this study systematically investigates the spatiotemporal evolution, network structure, and driving mechanisms of low-carbon technology collaborative innovation networks by integrating spatial statistical analysis, social network analysis, and the Geodetector method. The main conclusions are as follows.

First, the spatial distribution of low-carbon innovation activities exhibits pronounced agglomeration alongside dynamic evolution. Core cities such as Shanghai, Nanjing, Hangzhou, and Ningbo consistently function as major innovation hubs, while peripheral areas, particularly in northern Anhui, remain comparatively underdeveloped. Despite the continuous expansion of the network, significant regional disparities persist, reflecting the uneven spatial allocation of low-carbon innovation resources.

Second, the collaborative innovation network has evolved toward greater complexity, connectivity, and efficiency. Increases in network density, average degree, and clustering coefficient indicate more frequent interactions and stronger local clustering effects, while reductions in network diameter and average path length suggest enhanced efficiency in knowledge diffusion. Nevertheless, the persistence of a core–periphery structure dominated by a limited number of central cities points to potential risks of structural imbalance and path dependence.

Third, cities with high weighted degree centrality and betweenness centrality perform distinct yet complementary functions within the network. Cities with high weighted degree centrality exhibit strong resource agglomeration and participation intensity, serving as key innovation anchors. In contrast, cities with high betweenness centrality act as critical intermediaries that facilitate cross-regional knowledge flows. This dual structure underscores the joint importance of network embeddedness and brokerage roles in shaping the performance and resilience of the innovation system.

Fourth, the formation and evolution of the network are driven by a multidimensional set of factors, including economic scale, financial support, technological innovation capacity, and policy and institutional conditions. The Geodetector results further reveal significant interaction effects among these factors, indicating that low-carbon innovation is governed by a synergistic mechanism involving innovation actors, financial resources, and policy support. Such coupling not only strengthens network connectivity but also enhances the efficiency and sustainability of collaborative innovation.

### 7.2 Implications and recommendations

1. Hotspot cities such as Shanghai, Suzhou, and Wuxi should be encouraged to export talent, capital, and relevant technologies for low-carbon technological innovation to coldspot cities such as Bengbu, Bozhou, and Suzhou. Simultaneously, infrastructure investment and policy support should be increased to advance the establishment of low-carbon technology collaborative innovation platforms in the YRD, thereby elevating low-carbon technological innovation capabilities in coldspot areas. Cities such as Shanghai and Nanjing should be supported in establishing low-carbon technology cooperation and innovation centers for the YRD, thereby strengthening the hub functions of core cities.
2. Cross-provincial corporate cooperation mechanisms for low-carbon technological innovation should be established and improved. The intensity of cross-provincial collaborative innovation within the region should be increased, and administrative barriers should be reduced through measures such as cross-provincial credit support, streamlining cross-provincial cooperation processes, and establishing a special fund for low-carbon technological innovation cooperation and R&D in the YRD.
3. Universities, research institutes, and low-carbon technology innovation enterprises should be encouraged to form IUR alliances and promote data sharing and collaborative R&D in low-carbon technologies. Simultaneously, a low-carbon technological innovation trading platform in the YRD should be established to facilitate patent conversion and diffusion, enhancing the market efficiency of innovation outcomes.
4. To fully unlock the potential value of low-carbon technological innovation in the field of public health, it is recommended that all cities in the YRD integrate Health Impact Assessment into the planning and evaluation system for low-carbon innovation policies. When building innovation platforms or funding R&D projects, policymakers shall require an assessment of the expected health co-benefits brought by the policies. In addition, regular monitoring of air quality improvements and population health outcomes should be conducted in areas where low-carbon technologies are implemented and applied, so as to establish a feedback mechanism that links innovation activities with quantifiable health benefits.

### 7.3 Theoretical contributions

1. This study advances existing research by integrating spatial distribution and collaborative innovation networks within a unified analytical framework. While prior studies have typically examined the spatial agglomeration of innovation activities and inter-organizational networks separately, this paper demonstrates that these two dimensions are intrinsically interconnected and co-evolve over time. By focusing on low-carbon technology innovation enterprises as a specific analytical unit, the study reveals a dynamic process of mutual reinforcement between geographical proximity and network embeddedness, thereby enriching the theoretical understanding of innovation geography and offering more targeted insights for regional policy design (16–22).
2. This study addresses the limitations of traditional regional economic approaches, which often overlook the structural complexity and temporal dynamics of innovation networks. The findings deepen our understanding of the differentiated roles, formation mechanisms, and functional heterogeneity of “hub nodes” within innovation systems. Furthermore, the study highlights how institutional environments and structural conditions jointly shape network efficiency and inclusiveness, demonstrating that the strong interactive coupling between innovation actors and external contextual factors constitutes a core driving force behind network evolution. This contributes to the theoretical development of regional innovation systems and network-based perspectives on economic development (35–43).
3. This study makes an interdisciplinary theoretical advancement by conceptualizing low-carbon innovation networks as a form of “upstream health infrastructure.” It systematically establishes the linkage between technological collaboration, environmental outcomes, and public health implications. Specifically, the diffusion of low-carbon technologies through innovation networks can reduce carbon emissions, improve air quality, and subsequently lower population exposure to environmental health risks. From this perspective, unequal access to innovation networks may translate into disparities in environmental governance capacity and, ultimately, public health outcomes. By embedding health equity considerations into the analysis of technological and spatial systems, this study bridges the gap between innovation studies and public health research and proposes a novel analytical framework for understanding health inequalities through the lens of technological and spatial governance.

### 7.4 Research limitations and future directions

1. This study relies on patent data to identify low-carbon technology innovators and their collaborative innovation patterns, which may overlook nonpatent innovation activities such as academic publications. Future research should integrate data on R&D investments and collaborative publications among low-carbon technology innovators to comprehensively identify key players, subject to data availability.
2. This study primarily examines the YRD internally and lacks cross-regional comparisons with other urban clusters, such as the Beijing–Tianjin–Hebei or the Pearl River Delta. Future research should employ comparative analysis to derive more universally applicable conclusions and policy recommendations.
3. This study adopts four time points to capture the long-term evolution of innovation networks. Although these time nodes align with China’s Five-Year Plan cycles and effectively reflect the impacts of major policy adjustments, such a cross-sectional design may omit fine-grained annual dynamics, sudden turning points, and short-term structural fluctuations caused by exogenous shocks, limiting the refined depiction of the continuous evolutionary process of innovation networks.
4. The collaborative network in this paper is constructed based on patent co-application data, and the spatial location of each applicant is uniformly determined according to enterprise registered addresses. This standardized method can objectively and consistently characterize intercity collaborative relationships, yet it fails to fully reflect the spatial complexity of multi-location enterprises and cannot capture informal cooperation channels. Future research can incorporate data on enterprise branches and subsidiaries to achieve a more comprehensive measurement of collaborative linkages.

## Data Availability

The raw data supporting the conclusions of this article will be made available by the authors, without undue reservation.

## Footnotes

1
https://news.cctv.cn/2025/07/17/ARTIXofi13fYL0tJNIk9pcot250717.shtml

2
https://mee.gov.cn/xxgk2018/xxgk/xxgk13/202301/t20230112_1012533.html

3
https://www.gov.cn/gongbao/content/2019/content_5462503.htm?eqid=fb2c1bb00000dbd500000004647bee0f&f_link_type=f_linkinlinenote&flow_extra=eyJkb2NfcG9zaXRpb24iOjAsImRvY19pZCI6ImQ1M2Y3ZDBlOTEzYjViNDctZTM0ZTgwNWE2ZGU0NGEzZiIsImlubGluZV9kaXNwbGF5X3Bvc2l0aW9uIjowfQ%3D%3D

4
https://fg.suzhou.gov.cn/szfgw/stzc/202312/6855584184ac4d7289bcc411331e95a9/files/f8de9a4794f148779478392a3c345308.pdf?f_link_type=f_linkinlinenote&flow_extra=eyJpbmxpbmVfZGlzcGxheV9wb3NpdGlvbiI6MCwiZG9jX3Bvc2l0aW9uIjowLCJkb2NfaWQiOiIyNTkyNWVlMjg3ZWYzNDU5LTc2ZTEzMzU5OTZjNGM5MTgifQ%3D%3D

5
https://www.ah.gov.cn/public/1681/554149111.html

6
https://fgw.sh.gov.cn/fgw_zxwyta/20250513/4ae41c83d0c3425b834452ff96df187e.html
